# Interventions to Promote Physical Activity and Healthy Ageing: An Editorial

**DOI:** 10.3390/ijerph21091225

**Published:** 2024-09-18

**Authors:** Andy Pringle, Nicky Kime

**Affiliations:** 1 Clinical Exercise and Rehabilitation Research Centre, School of Sport and Exercise Science, University of Derby, Kedleston Road, Derby DE22 1GB, UK; 2 Bradford Institute for Health Research, Temple Bank House, Bradford Royal Infirmary, Bradford BD9 6RJ, UK

The Healthy Ageing Challenge aims for people to enjoy at least five extra healthy, independent years of life by 2035, while narrowing the gap between the experiences of the richest and poorest. Regular physical activity is important for healthy ageing, not only for maintaining health in midlife but also for maintaining health, independence and quality of life as people become older. Physical activity guidelines highlight the benefits and the importance of helping adults to adopt and maintain regular physical activity participation throughout the life course and not only in later life, and interventions and activities that support this aspiration are so important. We are delighted that this Special Issue features contributions that report both the impact and implementation of physical activity interventions for adults and older adults, as well as a range of studies on this topic [1]. Research highlights the importance of physical activity in maintaining functionality and retaining independence, in order that people can do all the things they want and need to do in their daily life [2,3]. One of the key features of the most recent UK physical activity guidelines is a greater emphasis being placed on activities that promote strength and balance [3]. Several of the papers in this Special Issue focus on this important topic, along with physical functioning [Contributions 1–4]. With those thoughts in mind, Davis and colleagues investigate the feasibility, psychosocial effects, influence, and perception of elastic band resistance balance training in older adults [Contribution 1]. Their pilot work also investigates the preferences, likes, and dislikes of older adults regarding the exercises. Understanding what works well and what works less well, and the reasons for this is important, as reiterated by Davis and several other studies in this collection. Indeed, holding dialogue with participants about their physical activity needs, preferences, likes, and dislikes is important in shaping the design and delivery of interventions. Devereux and colleagues remind us that some older adults in lower socioeconomic status (SES) areas are among the least active of all adult groups but are often absent from physical activity research. It is valuable that their study aimed to elicit important perspectives on the acceptability of physical activity from both the perspectives of older adults and physical activity providers [Contribution 5].

When we consider the providers of advice on physical activity, healthcare professionals (HCPs) are important agents for promoting physical activity to adults and older adults [3,4]. Cunningham and O’Sullivan explored HCPs’ knowledge, decision making, and routine practice of physical activity promotion with older adults [Contributions 6–7]. Less than a third of respondents had a clear plan on how to initiate discussions about physical activity in routine practice with older adults [Contribution 6]. Understanding the barriers that HCPs face when promoting physical activity is important. Research has investigated the role of doctors in promoting physical activity, including the barriers and facilitators they face in doing so [4]. Given the key role that HCPs play and, in some instances, their lack of preparedness for promoting a physically active lifestyle, insights are important in shaping activities which help support HCPs preparations in helping people to adopt physical activity [Contribution 6–7]. Although HCPs have been identified as key conduits for promoting physical activity [3,4], there are other individuals and agencies that also have a role to play. The importance of local community agencies in helping people start and keep physically active has long been identified [5]. Health interventions delivered through community-based agencies, foundations, and charities are an important part of the public health landscape, especially given a declining range of public services from some statutory bodies. One key feature of the offer made by community providers is connecting to people around their interests, including their leisure preferences. With this in mind, Cortnage’s study illustrates how to connect men to health improvement programmes through the power of football [Contribution 8]. Thinking about people’s active recreation preferences, it was, also, great to receive Iron’s ‘Parkinson Beats’ study, exploring people with Parkinson’s experience of cardio-drumming [Contribution 9]. In this spirit, Russell’s review, ‘“We Can Do This!”: The Role of Physical Activity in What Comes Next for Dementia’, focuses upon life with dementia and the role that physical activity can play within it [Contribution 10]. Indeed, several of our studies focus on adults with long-term conditions, including Wu’s case study, which explores interventions to improve the physical capability of older adults with mild disabilities [Contribution 3]. Finally, all our contributions to this Special Issue [1] provide valuable insights on undertaking research and evaluation within this context and provide learning to inform future investigations on this topic. This Special Issue brings together an eclectic mix of studies, including contributions from an international authorship [Contributions 2–4], which add to the insights provided in this body of work.

Nicky and I are pleased that early career researchers have also taken up the call to submit their work. Helping researchers to publish is something that we are passionate about. In this respect, we are grateful for the support our early career researchers received from more experienced colleagues, as well as the support offered by the editorial team at *IJERPH*. We are also grateful to every author who submitted to our call for papers [24 papers in total], especially to the authors whose papers were eventually included in other *IJERPH* Special Issues. We are grateful to our independent peer reviewers who provided feedback on the submissions, along with the thousands of readers who have already accessed this Special Issue. Finally, we thank all the people and professionals who took part in and supported the research featured in this Special Issue, which helped grow our understanding and knowledge of this important area of work.

We hope to follow this collection of works with a second edition, but for now, Nicky and I hope you enjoy reading this Special Issue.

## References

[1] Interventions to Promote Physical Activity and Healthy Ageing: Special Issue. *Int. J. Environ. Res. Public Health* (ISSN 1660-4601). https://www.mdpi.com/journal/ijerph/special_issues/aging_physical_intervention.

[2] Skelton D.A., Copeland R.J., Tew G.A., Mavroeidi A., Cleather D.J., Stathi A., Greig C.A., Tully M.A. Expert Working Group Working Paper UK Physical Activity Guidelines: Older Adults Working Group. Review and Recommendations for Older Adults (Aged 65+ Years). https://www.bristol.ac.uk/media-library/sites/sps/documents/cmo/older-adults-technical-report.pdf.

[3] Department of Health and Social Care (2018). Physical Activity Guidelines: UK Chief Medical Officers’ Report. A Report from the Chief Medical Officers in the UK on the Amount and Type of Physical Activity People Should Be Doing to Improve Their Health. London. https://www.gov.uk/government/publications/physical-activity-guidelines-uk-chief-medical-officers-report.

[4] Vishnubala D., Iqbal A., Marino K., Whatmough S., Barker R., Salman D., Bazira P., Finn G., Pringle A., Nykjaer C. (2022). UK Doctors Delivering Physical Activity Advice: What Are the Challenges and Possible Solutions? A Qualitative Study. Int. J. Environ. Res. Public Health.

[5] Goodman C., Davies S., Tai S.S., Dinan S., Iliffe S. (2007). Promoting older peoples’ participation in activity, whose responsibility? A case study of the response of health, local government and voluntary organizations. J. Interprof. Care.

